# Microalgal Astaxanthin: From Metabolic Regulation to Industrial Biomanufacturing for a Sustainable Bioeconomy

**DOI:** 10.3390/life16101631

**Published:** 2026-09-29

**Authors:** Yiwen Chen, Gerui Yin, Yuxue Chen, Haiyan Zhai, Jiabao Yang, Jilong Zhao, Mengchi Wang, Fantao Kong

**Affiliations:** MOE Key Laboratory of Bio-Intelligent Manufacturing, School of Bioengineering, Dalian University of Technology, Dalian 116024, China; 32547067@mail.dlut.edu.cn (Y.C.); geruiyin@mail.dlut.edu.cn (G.Y.); chenyuxue@mail.dlut.edu.cn (Y.C.); zhaihaiyandut@mail.dlut.edu.cn (H.Z.); yangjiabao@mail.dlut.edu.cn (J.Y.); 32347024@mail.dlut.edu.cn (J.Z.); wangmengchidut@mail.dlut.edu.cn (M.W.)

**Keywords:** astaxanthin, nutraceuticals, green biorefinery, microalgae, *Haematococcus lacustris*, sustainable bioeconomy

## Abstract

Astaxanthin is a ketocarotenoid with potent antioxidant properties. It exhibits higher antioxidant activity than several other carotenoids and has potential applications in cosmetics, nutraceuticals, and pharmaceuticals. Microalgae are the primary producers in aquatic ecosystems. They are also regarded as sustainable and promising chassis for a sustainable bioeconomy due to their efficient photosynthesis and ability to convert CO_2_ into valuable products. However, the current large-scale production of astaxanthin from microalgae is still limited due to critical bottlenecks, including unclear regulatory mechanisms of astaxanthin biosynthesis, suboptimal strain performance and low product titers, resulting in the high cost of phototrophic cultivation and downstream processing. This review summarizes recent studies on the metabolic regulation of astaxanthin biosynthesis and technological advances in industrial biomanufacturing, highlighting how integrated strategies can enable a sustainable bioeconomy. We propose a conceptual and modular framework for a green astaxanthin intelligent manufacturing system, which integrates breeding and metabolic regulation, large-scale cultivation process optimization, green separation, extraction and application. This review would be helpful for upgrading the industrial development of microalgal astaxanthin to the strategic level of supporting global climate change responses, protecting ecological resource security, and promoting the green industrial revolution.

## 1. Introduction

**Figure 1:**
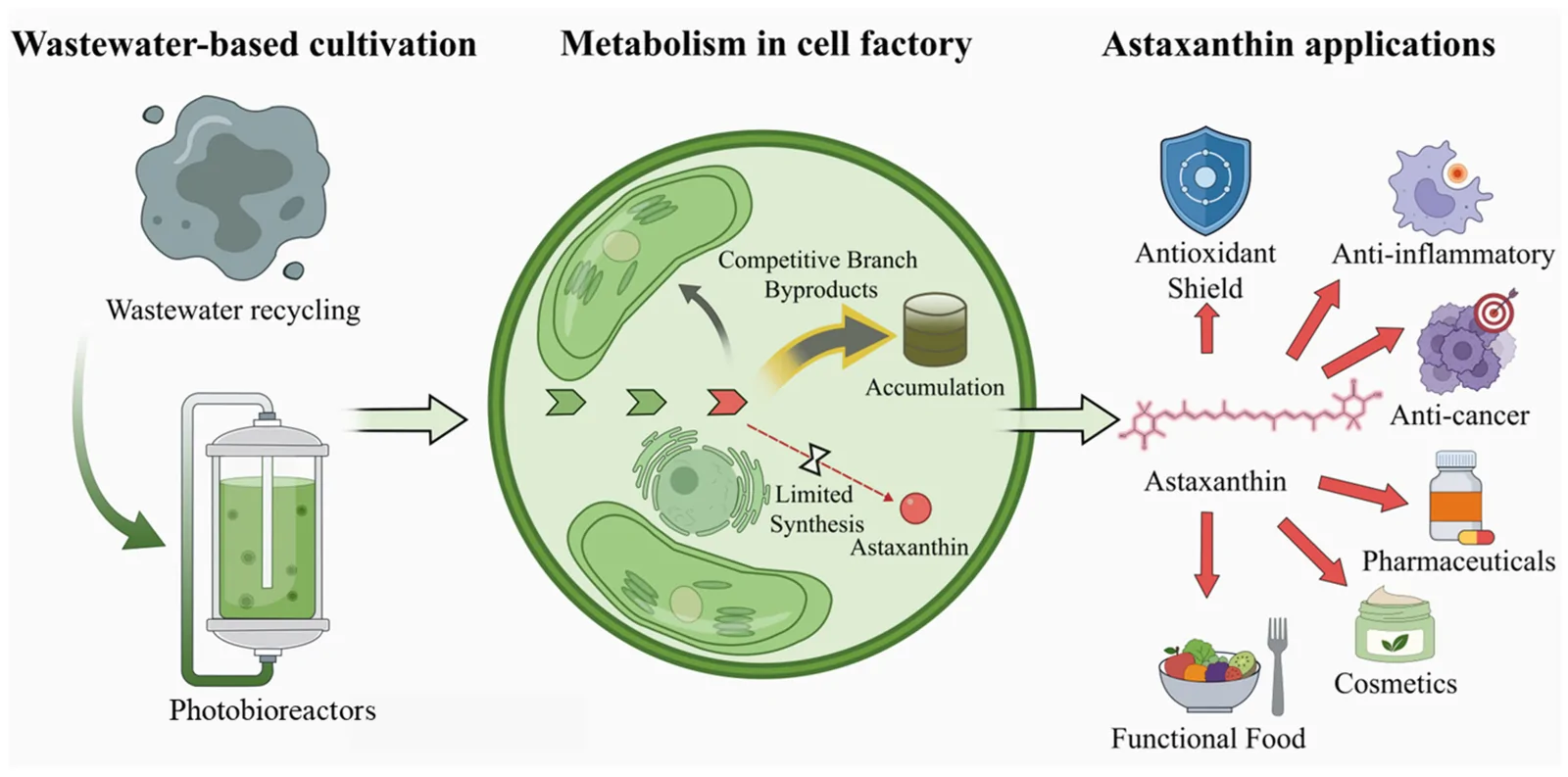
Schematic illustration of microalgae-based astaxanthin production integrated with wastewater treatment, and its downstream applications in pharmaceuticals, cosmetics and nutraceuticals.

Astaxanthin (3,3′-dihydroxy-β, β-carotene-4, 4′-dione) is an orange-red ketocarotenoid, which is widely utilized in biomedicine, functional foods, animal feed and cosmetics, due to its potent antioxidant activity [1]. Currently, chemically synthesized astaxanthin remains the dominant form in the global market. However, naturally derived astaxanthin (NAT-AX) exhibits superior biological activity compared to its synthetic counterpart (SYN-AX). For instance, antioxidant experiments have demonstrated that NAT-AX exhibits 14-fold to 90-fold greater antioxidant activity than SYN-AX [2]. Accordingly, natural astaxanthin is preferable for use as a functional dietary supplement, offering multiple health benefits to the human body. Its antioxidant, anticancer, and anti-inflammatory activities endow it with substantial developmental value in the domains of pharmaceuticals, nutraceuticals, and functional foods (Figure 1) [3]. Driven by escalating global demand, astaxanthin biosynthesis has emerged as a key frontier in industrial biotechnology and nutraceutical development [2,3]. Among astaxanthin producers, microalgae are recognized as the optimal production system for astaxanthin biosynthesis, yielding astaxanthin with significantly greater bioactivity than that obtained from yeast or higher plants [3]. However, current microalgal astaxanthin production remains insufficient to meet global market demand. Confronting this shortfall, previous studies have mainly focused on engineering model microalgae or redirecting their metabolic pathways through applied environmental stress to boost biosynthetic yields. Currently, the algal strains examined for astaxanthin production mainly include *Haematococcus lacustris* (formerly *H. pluvialis*), *Chlamydomonas reinhardtii*, and *Chromochloris zofingiensis* [4]. Substantial efforts have been dedicated to optimizing astaxanthin yields in these algal species (Table 1). For instance, heterologous expression of a prokaryotic lycopene β-cyclase in the eukaryotic microalga *C. reinhardtii* enhanced the yield of the astaxanthin precursor β-carotene by 2.5-fold, which may provide a promising strategy for boosting astaxanthin production in *C. reinhardtii* [5]. Environmental stress represents another effective strategy for promoting astaxanthin accumulation. Notably, *H. lacustris* can produce up to 7.60 mg/L astaxanthin under high-salinity or nitrogen-depleted conditions [6]. This approach has also been extended to *C. zofingiensis* via a two-stage heterotrophic cultivation process, achieving simultaneous high biomass and astaxanthin productivity [7].

Despite extensive genetic, environmental, and cultivation-based optimization strategies applied to *H. lacustris*, *C. reinhardtii*, and *C. zofingiensis*, significant improvements in astaxanthin yield remain elusive, primarily constrained by the inherent bottlenecks of their endogenous metabolic pathways. The global astaxanthin market was estimated to reach USD 4.27 billion by 2033, with demand for natural astaxanthin currently exceeding supply [8]. However, *H. lacustris*, the benchmark natural producer, accumulates astaxanthin at 3–5% of dry biomass but reaches biomass densities of only 5–10 g L^−1^, whereas *C. zofingiensis* achieves biomass concentrations of 100–220 g L^−1^ under heterotrophic fermentation but with much lower astaxanthin content (0.1–0.5% DW) [9]. The major bottlenecks are the metabolic trade-offs between endogenous precursor (e.g., GGPP) availability and cofactor (e.g., NADPH and ATP) homeostasis, coupled with a lack of coordinated interplay between stress signaling and antioxidant responses [10]. Moreover, transcription factors (e.g., NAC and AP2/ERF) significantly correlate with astaxanthin-biosynthesis-related genes in *C. zofingiensis*, serving as regulatory targets in astaxanthin accumulation [11]. The first issue arises from competitive branch pathways or intermediate product diversion within the astaxanthin biosynthetic pathway, which depletes metabolic precursors and restricts astaxanthin yield (Figure 1) [4]. To address this, multi-gene combinatorial expressions in engineered strains were generated [12]. For example, the sequential expression of *CrBKT* (β-carotene ketolase), *PacrtB* (phytoene synthase), and *CrCHYB* (β-carotene hydroxylase) in *C. reinhardtii*, combined with high-cell-density cultivation, yielded an astaxanthin titer of 23.5 mg/L in the transformants [13]. The second limitation concerns the application of environmental stressors. Although these stressors can activate ROS-mediated signaling to upregulate astaxanthin biosynthetic genes, they also intensify competition for carbon flux redistribution. This metabolic trade-off reduces pathway efficiency and impairs biomass accumulation, thereby limiting astaxanthin yield [14]. Therefore, developing strategies to fine-tune stress-induced metabolic flux is critical [15]. The inherent constraints of native metabolism and the limitations imposed by stress induction are fundamentally linked. Therefore, a multidimensional strategy that integrates genetic engineering with precise exogenous stimulation is essential to maximize astaxanthin biosynthesis in microalgae [16].

Water consumption represents yet another critical bottleneck limiting the sustainability of large-scale astaxanthin production from microalgae. Replacing freshwater with wastewater for the cultivation of astaxanthin-accumulating strains, including *H. lacustris* and *C. zofingiensis*, represents a viable pathway to reduce freshwater dependency in microalgal biorefining, while converting nutrient rich waste into valuable bioproducts, subject to product safety validation and regulatory compliance (Figure 1) [17]. This approach leverages the robust nutrient-assimilating capacity of these algae, which are known to efficiently take up organic carbon, nitrogen and phosphorus at high concentrations in industrial wastewaters. Through dynamic reprogramming of intracellular carbon and nitrogen fluxes, *H. lacustris* and *C. zofingiensis* can efficiently redirect wastewater-derived nutrients toward high-value biomass components, including astaxanthin [18]. Consequently, *H. lacustris* and *C. zofingiensis* are regarded as candidates for integrated wastewater valorization and astaxanthin production [19]. In aquaculture wastewater treatment, *H. lacustris* can remove 98% of total nitrogen and 91% of total phosphorus while accumulating >10 mg/g astaxanthin, representing a paradigm of synergistic pollution remediation and resource recovery [20]. It was reported that *C. zofingiensis* cultivated autotrophically in two-stage photobioreactors containing 2.5% palm oil mill effluent produces 2.7 mg/L astaxanthin, directly converting high-strength organic waste into terpenoid pigments [21]. The feasibility of this wastewater-to-product pathway relies on the intrinsic photosynthetic autotrophic metabolism of microalgae [22], which enables the simultaneous assimilation of wastewater organics and atmospheric CO_2_, thereby coupling astaxanthin biosynthesis [23]. Thus, maximizing this synergy requires a precise understanding and engineering of photosynthetic carbon fixation kinetics in large-scale photobioreactors [24]. In a pilot-scale study, *H. lacustris* cultivated in a 40 L tubular photobioreactor under a carbon sequestration regime achieved an astaxanthin yield of 68.9 mg/L over 25 days, demonstrating that targeted optimization of carbon assimilation kinetics can enhance both astaxanthin productivity and carbon capture [25].

This review summarizes recent advances in the development of novel high-yield strains, with particular emphasis on genetic engineering strategies that overcome the intrinsic constraints of conventional models. It further outlines a systems-level engineering framework for astaxanthin overproduction in microalgae, centered on the rational redesign of global metabolic networks and precise dynamic control of carbon flux, overcoming the inefficiencies inherent in single-gene interventions. Additionally, it evaluates the industrial translation of microalgal astaxanthin production within a green biorefinery paradigm, integrating pigment recovery, wastewater treatment, and CO_2_ capture to enable circular bioeconomy operations. Taken together, this review establishes a robust conceptual and practical foundation for sustainable astaxanthin manufacturing, supporting the shift toward scientifically rigorous and industrially scalable bio-based production systems.

This review was constructed through a systematic search of the Web of Science Core Collection and PubMed databases, with emphasis on research published in the last ten years. The search strategy combined terms related to: (i) the target compound, including “astaxanthin”, “ketocarotenoid” and “natural astaxanthin”; (ii) production hosts, including “microalgae”, “microalgal”, “*Haematococcus lacustris*”, “*Chromochloris zofingiensis*”, “*Chlamydomonas reinhardtii*”, “*Dysmorphococcus globosus*” and “*Cyanidioschyzon merolae*”; and (iii) thematic areas, including “biosynthesis”, “metabolic engineering”, “CRISPR-Cas”, “strain screening”, “mixotrophic cultivation”, “light quality”, “artificial intelligence”, “machine learning”, “wastewater”, “CO_2_ capture”, “green extraction”, and “biorefinery”. The Boolean operators AND/OR were used to combine these terms.

## 2. Screening of Optimized Algal Strains for High-Yield Astaxanthin Production

### 2.1. The Development of Astaxanthin Production in Extremophilic Microalgae

Extremophilic microalgae have recently garnered considerable attention due to their intrinsic traits, including acid tolerance and thermophily. These characteristics not only provide natural resilience against biological contamination in open pond systems but also present a promising avenue for addressing the productivity constraints of conventional strains [26]. For example, heterologous expression of *CrBKT* and *CrCHYB* from *C. reinhardtii* in the thermoacidophilic red alga *C. merolae* enabled astaxanthin accumulation (0.45–0.69 wt %) without compromising phycocyanin production, thereby establishing extremophilic algae as a robust chassis for astaxanthin production [27]. However, when the wild-type and *CrBKT*-*CrCHYB*-expressing strains were cultured in 400 mL cultures using a suite of photobioreactors, the transformants showed slightly lower optical density, cell density, and biomass than the parental strain. Nevertheless, both the transformed and parental *C. merolae* strains were still able to grow under these conditions, with the transformants accumulating the ketocarotenoid product. More recently, the zinc-tolerant extremophile *D. globosus* has emerged as another promising chassis for astaxanthin production. The optimal Zn^2+^ level in the culture medium enhanced astaxanthin and other pigment production. Specifically, when Zn^2+^ was supplied at 45.5 mg L^−1^, *D. globosus* ZY24 yielded 0.31 mg g^−1^ astaxanthin [28].

### 2.2. Screening and Culturing Red Motile Cells Under High-Light Conditions

The thick cell wall represents a critical bottleneck in industrial-scale astaxanthin production from microalgae [29]. This adaptive defense mechanism not only complicates downstream extraction but can also offset the economic gains of improved productivity. In wild-type *H. lacustris*, this characteristic has given rise to two persistent limitations that have hindered industrial development for decades [30]. Firstly, oxidative stress induces differentiation into dormant cysts characterized by rigid, tri-layered, acid-resistant walls, which entrap astaxanthin and require energy-intensive mechanical disruption for recovery. Secondly, the inherent coupling between astaxanthin biosynthesis and cell wall thickening has restricted astaxanthin titers to 20 mg/L in the wild type [28]. A recent breakthrough circumvented this barrier via targeted screening, isolating the astaxanthin-rich motile strain RMS10. The mutant strain RMS10, derived from the parental strain NBU489, exhibits improved biomass and astaxanthin content. Remarkably, RMS10 facilitates astaxanthin release without the need for external cell disruption, producing 44.6 mg/g, a 53.3% increase relative to NBU489, and providing an alternative approach to circumventing the cell wall bottleneck [31].

## 3. Metabolic Engineering of Astaxanthin Pathway for Astaxanthin Production

### 3.1. Astaxanthin Biosynthesis Pathway in Microalgae

**Figure 2:**
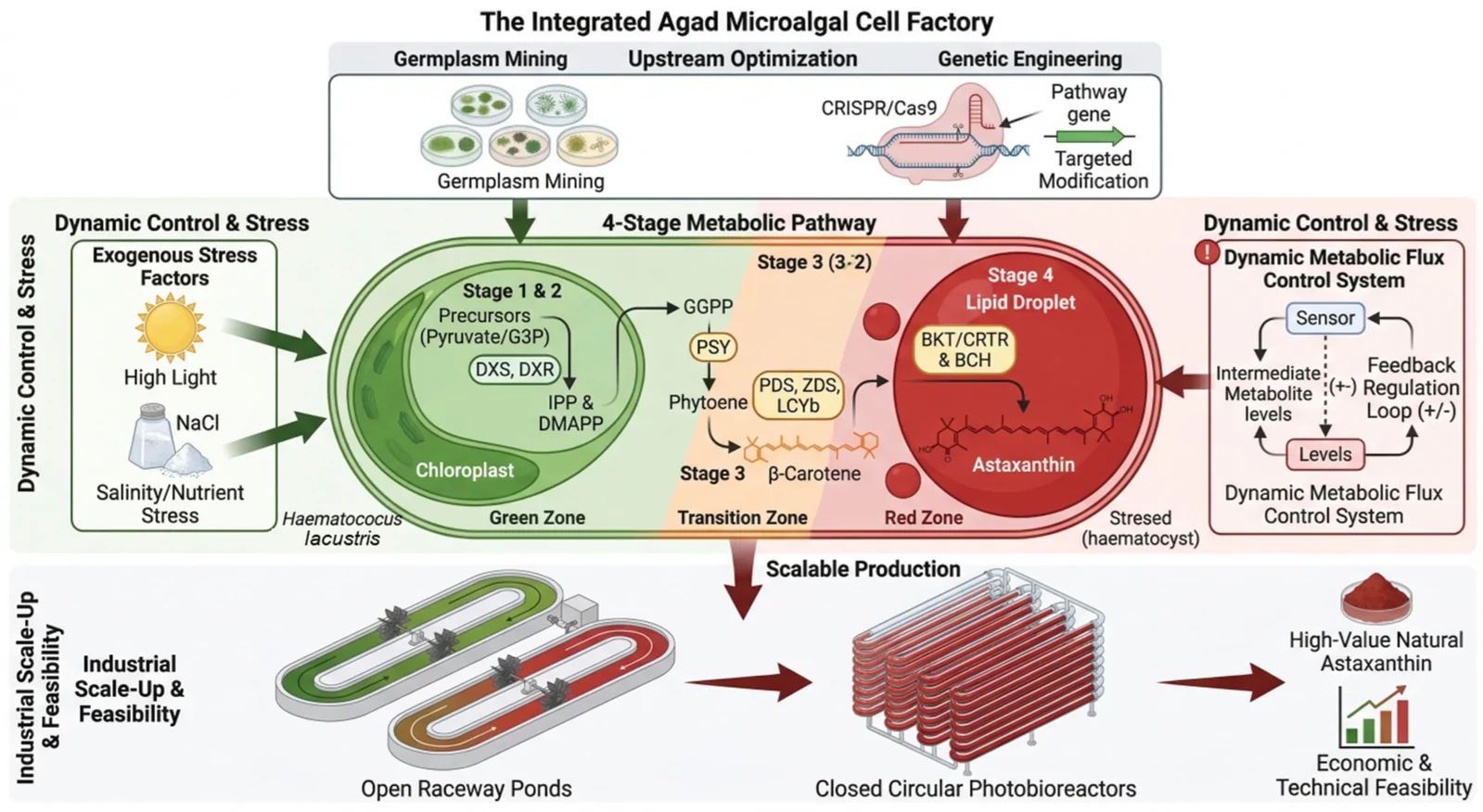
Systems−level strategy for microalgal astaxanthin biomanufacturing, integrating strain selection, metabolic pathway regulation, process optimization, and large−scale cultivation to achieve high productivity and industrial viability. CRISPR-Cas, clustered regularly interspaced short palindromic repeats (CRISPR) and CRISPR-associated proteins; PSY, phytoene synthase; PDS, phytoene desaturase; IPP, isopentenyl pyrophosphate; DMAPP, dimethylallyl pyrophosphate; DXS, deoxyxylulose 5-phosphate synthase; DXR, deoxyxylulose 5-phosphate reductoisomerase; GGPP, geranylgeranyl diphosphate; ZDS, ζ-carotene desaturase; Z-ISO, 15-cis-ζ-carotene isomerase; LCYB, lycopene β-cyclase; LCYE, lycopene ε-cyclase; BKT, β-carotene ketolase; CrtR, β-carotene hydroxylase.

Astaxanthin biosynthesis proceeds through four sequential stages that are spatially partitioned in microalgal cells (Figure 2). Central carbon metabolism in the cytosol and plastid generates pyruvate, acetyl-CoA and glyceraldehyde-3-phosphate, which feed the plastid-localized methylerythritol phosphate (MEP) pathway. In the plastid, DXS and DXR catalyze the committed steps of the MEP pathway to produce IPP and DMAPP. IPP and DMAPP are condensed by GGPP synthase and PSY to phytoene; subsequent desaturation/isomerization by PDS, Z-ISO, ZDS and CRTISO yields all-*trans*-lycopene, which is cyclized by LCYB and LCYE to β-carotene and α-carotene, respectively. β-carotene, the direct precursor of astaxanthin, is converted by BKT and CrtR-B at the plastid membrane/envelope, after which astaxanthin is esterified and sequestered in plastoglobuli or cytosolic lipid droplets [11]. Thus, the pathway is not a single cytosolic cascade but a plastid-centered network with downstream storage in lipid droplets. This compartmental architecture is essential for interpreting flux control: MEP/GGPP/PSY steps determine plastidial precursor supply, LCYB/LCYE competition determines lycopene partitioning, and BKT/CrtR-B localization and activity determine the efficiency of β-carotene conversion [32,33].

High light intensity is known to significantly upregulate *PSY* expression. In synergy with nitrogen starvation, the PSY substrate GGPP can be utilized more efficiently, promoting the accumulation of phytoene, a key intermediate in astaxanthin synthesis [34]. Subsequently, phytoene undergoes dehydrogenation, isomerization, and multiple desaturation reactions catalyzed by the conserved PDS-Z-ISO-ZDS-CRTISO enzyme cascade to form pro-lycopene, which is then converted to all-trans-lycopene via CRTISO-mediated configuration isomerization [35]. Lycopene represents a critical branch point in the carotenoid biosynthetic pathway. In microalgae, lycopene is diverted by lycopene β-cyclase and ε-cyclase toward the synthesis of various carotenoids, including β-carotene and α-carotene, a crucial step in astaxanthin biosynthesis [36]. Lycopene β-cyclase catalyzes the β-cyclization at the termini of lycopene: the first cyclization yields γ-carotene (containing one β-ring), and the second cyclization occurs at the other end, ultimately producing β-carotene with two β-rings [37].

β-carotene is the direct precursor of astaxanthin biosynthesis [38]. The single-end ε-cyclization of lycopene mediated by lycopene ε-cyclase, in cooperation with the β-cyclization reaction on the opposite side catalyzed by lycopene β-cyclase, specifically generates α-carotene [39]. α-carotene is a precursor of other carotenoids such as lutein, not a direct precursor of astaxanthin [40]. Therefore, the effective diversion and utilization of lycopene are key factors affecting the carotenoid composition in microalgae [41]. When lycopene is diverted to β-carotene, the conversion from β-carotene to astaxanthin is completed through the synergistic action of β-carotene ketolase (BKT) and β-carotene hydroxylase (CrtR-B) [42]. This process is regulated by environmental stress, which can prompt microalgae to enter the “red stage” to accumulate a large amount of astaxanthin and avoid oxidative damage to cells [43]. For example, under high-light conditions, to reduce the damage to PSII proteins, the expression of genes encoding BKT, CrtR-B, and lycopene β-cyclase in *H. lacustris* is significantly upregulated to produce more astaxanthin [44].

### 3.2. Astaxanthin Pathway Rewiring Using Advanced Genetic Engineering Technologies

Optimizing the expression of astaxanthin biosynthetic genes and the related key enzymes, through co-expression strategies or targeted genetic engineering, is essential for reshaping the astaxanthin biosynthetic network and achieving efficient biomanufacturing. For example, the prokaryotic microalga *Synechococcus* sp. PCC 11901 was engineered with *BKT* and *CrtZ co*-expression to redirect metabolic flux, achieving astaxanthin production of 10 mg·L^−1^·d^−1^. This performance significantly surpasses conventional *H. lacustris* cultures in tubular or bubble-column photobioreactors, and is also superior to other heterologous systems such as *Synechocystis* sp. PCC 6803 (2.8 mg·L^−1^·d^−1^) and *C. reinhardtii* (6.96 mg·L^−1^·d^−1^) [12]. Furthermore, a synergistic enhancement system combining genome editing with environmental regulation was developed in *C. reinhardtii*. The *Δzl-bkt Chlamydomonas* strain was generated by simultaneous knockout of *LCYE* and *ZEP* (zeaxanthin epoxidase). This strain produced up to 2.13 mg/L astaxanthin, a yield significantly higher than that of the parental control under low-light conditions [38].

Astaxanthin production can be enhanced by eliminating negative regulatory factors and blocking competing pathways. For example, CRISPR-Cas-mediated knockout of negative regulators enables the targeted redirection of carbon flux toward astaxanthin biosynthesis [45]. In microalgae, the precursors for astaxanthin, IPP and DMAPP, are primarily derived from the methylerythritol phosphate (MEP) pathway. Disruption of negative regulatory genes within this pathway can boost precursor supply, which is critical for astaxanthin synthesis [46]. Beyond precursor availability, the activity of key rate-limiting enzymes acts as a major checkpoint in the biosynthetic pathway [47]. To achieve full conversion of β-carotene to astaxanthin and minimize intermediate accumulation, the CRISPR-Cas9 system can be applied to knock out the lycopene ε-cyclase (*LCYE*) gene, redirecting lycopene flux through the lycopene β-cyclase (*LCYb*)-mediated branch. This creates a metabolic environment conducive to the expression of optimized *BKT* and *CrtR-B* variants in engineered strains [48]. Subsequently, through mutant library construction and high-throughput screening, mutants with improved astaxanthin accumulation can be obtained [49]. Therefore, a combination strategy of negative regulator knockout and competitive pathway blockade can enhance astaxanthin accumulation.

In addition to carbon flux redistribution, overcoming the metabolic bottlenecks imposed by subcellular compartmentalization represents another core direction for optimizing astaxanthin synthesis. The biosynthesis of astaxanthin in microalgae is a highly coordinated process involving five major pathways: glycolysis, the methylerythritol 4-phosphate (MEP) pathway, geranylgeranyl pyrophosphate (GGPP) synthesis, carotenoid synthesis, and ketocarotenoid synthesis [50]. Enzymes participating in these pathways are typically distributed across distinct subcellular compartments—including the cytoplasm, endoplasmic reticulum (ER), and mitochondria. If key enzymes are not properly targeted to specific organelles or compartments, restricted diffusion between enzymes and substrates can impede metabolic flux, cause cross-interference with other cytoplasmic pathways, and ultimately compromise the efficiency of astaxanthin production [51,52,53].

## 4. A Systems Biology Strategy for Astaxanthin Accumulation

### 4.1. Culture Mode Optimization for Carbon Flow Reprogramming

Achieving efficient and large-scale astaxanthin production also lies in establishing a dynamic cultivation regulatory system, thereby enhancing the controllability and predictability of the production process (Figure 2). Metabolic flux optimization studies have revealed that microalgae can adopt three growth modes, autotrophic, heterotrophic and mixotrophic, among which the mixotrophic mode offers superior control over carbon flux distribution [54]. This mode integrates the benefits of both photoautotrophy and heterotrophy, allowing microalgae to utilize light energy, CO_2_, and organic carbon sources simultaneously, resulting in a synergistic effect [55]. Under mixotrophic conditions, using acetate as the organic carbon source and fine-tuning the ratio of organic to inorganic carbon (CO_2_) activate the glyoxylate shunt. This shunt bypasses the two decarboxylation steps of the TCA cycle, reducing carbon loss while establishing a dynamic synergy with the TCA cycle. This feedback-based adjustment of carbon metabolic flux enhances carbon turnover efficiency and enriches the precursor pool for astaxanthin biosynthesis [56].

### 4.2. Light Quality Regulation of Metabolic Remodeling

Light quality regulation has emerged as a powerful strategy for enhancing astaxanthin production. Different wavelengths can diferentially modulate carbon uptake, central carbon metabolism, and carbon partitioning in microalgae, with blue light being the most effective in inducing astaxanthin accumulation [57]. Under optimal blue light induction, the metabolic network directing astaxanthin synthesis exhibits a characteristic “one inhibition, two activations” regulatory pattern: the TCA cycle is suppressed to promote carbon conservation, the MEP pathway is activated to enhance the supply of key intermediates, and the pentose phosphate pathway (PPP) is upregulated to provide the reducing power (NADPH) and energy (ATP) required for astaxanthin biosynthesis. This coordinated multi-pathway regulation significantly enhances astaxanthin yield in microalgae [58].

### 4.3. Artificial Intelligence-Driven Cultivation Process Optimization

Although empirical interventions can partially optimize metabolic flux, astaxanthin biosynthesis is a highly nonlinear process involving the coupling of multiple parameters, making traditional trial and error approaches insufficient for identifying global optima. Consequently, a systematic modeling and prediction framework has become essential for overcoming this bottleneck [59]. Currently, kinetic models, response surface methodology (RSM), artificial neural networks (ANNs), and genome-scale metabolic models (GEMs) have been widely employed to simulate and predict microalgal growth and astaxanthin production [60]. Kinetic models, including the Andrews Haldane, Monod, Droop, and Luedeking Piret models, can describe photosynthetic rates, nutrient uptake, and cell division, thereby predicting biomass accumulation and astaxanthin titers [61]. When key input parameters such as light intensity, nutrient concentration, temperature, and pH are provided, these models have been experimentally validated and exhibit high predictive accuracy (e.g., R^2^ > 0.95) [62].

A major challenge, however, lies in the complex nonlinear interactions among multiple input parameters, which jointly affect several response variables. Effectively extracting key regulatory mechanisms from large experimental datasets and establishing reliable input–output relationships have become bottlenecks for accurately predicting microalgal growth dynamics and identifying optimal astaxanthin accumulation conditions [63]. To address this, RSM can be employed to generate response surfaces from experimental data, while ANNs leverage their powerful nonlinear mapping and generalization capabilities to predict growth and astaxanthin production [64]. For example, RSM and ANN models have demonstrated excellent predictive performance for carbohydrate accumulation in microalgae under different experimental conditions, achieving correlation coefficients of 0.9873 and 0.9959, respectively [65]. However, kinetic models, RSM, and ANNs are inherently limited in their ability to analyze the key steps of astaxanthin biosynthesis at a global level. Achieving a system-level understanding of the entire process requires the insights provided by genome-scale metabolic models (GEMs). By applying constraint-based methods such as flux balance analysis (FBA), GEMs can predict maximum biomass and astaxanthin yields under various nutrient limitations, light conditions, or genetic perturbations, while also identifying potential metabolic bottlenecks. This enables the design of novel metabolic pathways to enhance target product formation [60,66].

Integrating the strengths of RSM and ANNs, both rooted in machine learning (ML), with GEMs creates an embedded cross framework: a ternary bidirectional vector metabolic model. This integrative approach offers two major advantages. Firstly, it improves the accuracy of metabolic network reconstruction using multi-omics data, uncovers hidden regulatory mechanisms, and predicts missing reactions or enzyme functions. Secondly, it streamlines the manual curation of GEMs through ML, substantially reducing the need for labor-intensive intervention. Furthermore, by incorporating metabolic flux and other multi-omics data, this framework can guide strategy optimization, enabling precise global regulation of astaxanthin biosynthesis [67]. The integration of ML and GEMs, including other kinetic models, offers a promising route toward predictive and adaptive control of astaxanthin production. Nevertheless, this integrated framework remains prospective for microalgal astaxanthin and requires validation with high-quality, astaxanthin-specific datasets before industrial deployment.

## 5. Green Biorefinery of Microalgae-Derived Astaxanthin

### 5.1. Microalgal Astaxanthin Production Coupled with Wastewater Recycling

Although the industrialization of microalgae astaxanthin production depends on strain improvement, pathway reengineering, and system regulation, achieving environmentally friendly and low-cost sustainable production is the primary challenge for industrialization [68]. Agricultural and food wastewater systems, as one of the largest freshwater consumers globally, discharge nutrient-rich wastewater with low toxicity and significant resource potential [69]. Due to the superior wastewater treatment and bio-refining capabilities of microalgae, they can be directed to convert organic matter in wastewater into high-value products such as astaxanthin, subject to product safety validation and regulatory compliance. Utilizing nutrient-rich wastewater for large-scale microalgae cultivation to achieve pollutant purification and carbon resource recovery is a feasible green closed-loop solution [70]. For instance, in the Kingsburgh WWTP’s circular pond under natural conditions, an algal pond system was used for production. By monitoring feedback parameters and integrating ANNs for prediction, a complete system was established. The results demonstrated that the purified microalgae community had an efficient removal capacity for nitrogen and phosphate, and its cost for producing biofuels was lower than that of photobioreactor systems [71]. This verified the technical and economic feasibility of large-scale processes. However, whether these strategies can be applied to industrial astaxanthin production remains unknown, as their industrial implementation requires product safety assessment and compliance with regulatory requirements.

Besides circular algal ponds, open raceway ponds have also been proven to be a feasible cultivation system for large-scale microalgal production (Figure 2). In the Middle East, the extremophile strain *Chlorella sorokiniana* NIES-2169, which is heat-tolerant, was selected as the target algal species. A staged stepwise amplification cultivation strategy was adopted, and a harvest density of 1.0 g-DW L^−1^ was achieved in a 420 m^2^ large raceway pond [72]. This verified the feasibility of large-scale cultivation in open systems.

### 5.2. Green Extraction and Biorefining Technology

After achieving large-scale cultivation of astaxanthin-rich microalgal biomass, establishing an efficient and environmentally friendly extraction process for astaxanthin separation and purification becomes a critical step toward industrial application. Emerging green cell disruption technologies for extracting high-value compounds from microalgae include ionic liquids, deep eutectic solvents, surfactants, switchable solvents, high-voltage electrical pulse discharge, explosive decompression, compression expansion, plasma treatment and ozone treatment [73]. Among these, supercritical fluid extraction (SFE) is a representative technique that operates above the critical point, eliminating the distinction between gas and liquid phases [74]. CO_2_ is the primary supercritical solvent used for astaxanthin extraction from microalgae. SFE CO_2_ is well suited for industrial-scale applications due to its high selectivity and recyclability [75]. However, current green extraction processes may exhibit significant variations in effectiveness across different microalgal species, and some technologies are associated with high energy consumption and costs. Therefore, developing a more universal, energy-efficient, and cost-effective green extraction process is an inevitable requirement for future green biomanufacturing.

**Table 1:** Representative studies of microalgae-based astaxanthin production.

Microalgal Species	Engineering Strategy	Production Strategy	Production Scale	Yield	Productivity	Content	References
*H. lacustris*	/	Activating the mevalonate pathway under high-salt and nitrogen-deficiency conditions	Small-scale in laboratory	7.51 mg·L^−1^	0.29 g·m^−2^·d^−1^	/	[76]
	/	Cells are fixed by an alginate hydrogel membrane.	5-L glass conical flask	26.3 mg/d	256.3 mg⋅m^−2^⋅d^−1^	/	[77]
	/	Bacterial cellulose immobilization technology	Screen panel photobioreactor (0.3 m × 0.2 m × 0.04 m)	/	343.3 mg⋅m^−2^⋅d^−1^	40.23 mg/g	[78]
	/	Under high-light and nitrogen starvation conditions	1-L flat-panel photobioreactor	/	0.29 g·m^−2^·d^−1^	3.21% DW	[79]
	/	Constructing a PBR-LGP-PPR array hybrid system	PLPA Hybrid System (10^5^ L)	/	7.364 mg·L^−1^·d^−1^	/	[80]
	/	Two-stage calcium ion regulation	100-L polymeric photobioreactor	/	74.21 mg·L^−1^	/	[81]
	/	Rotating algal biofilm system	Rotating biofilm system (4 L)	/	204.00 mg⋅m^−2^⋅d^−1^	/	[82]
*C. reinhardtii*	Overexpressing CRT and BKT	Light induction and salt-induced stress conditions	Liquid shake flasks	/	/	1.47 mg·g^−1^	[83]
	Optimized the CrBKT sequence	Cultivated with 3% CO_2_ bubbling at 500–3000 µmol photons/m^2^/s in mixotrophic conditions	Liquid shake flasks	/	2.07 mg·L^−1^·d^−1^	/	[84]
*C. zofingiensis*	/	Mixed nutrition pre-cultivation combined with supplementary feed	Fermenter (20 L)	/	111.00 mg·L^−1^·d^−1^	/	[85]
	Addition of exogenous gibberellin	Fed-batch heterotrophic culture methodology	Fermenter (7.5 L)	0.34 g·L^−1^	/	/	[86]
	Flavonoids (rutin) stimulation	Resuspension in nitrogen-free medium with rutin (1.0 mM)	Bubble column bioreactor (3.5 cm diameter, 70 cm height)	/	3.44 mg·L^−1^·d^−1^	6.89 mg·g^−1^	[87]
	/	Salicylic acid induction	Erlenmeyer flask (100 mL)	0.175 g·L^−1^	/^−1^	/	[88]

Note: “/” indicates not applied. PBR-LGP-PPR, photobioreactor (PBR)-light guide panel (LGP)-PBR array (PLPA); CRT, β-carotene hydroxylase; DW, dry weight; BKT, β-carotene ketolase; LCYE, lycopene ε-cyclase; ZEP, zeaxanthin epoxidase.

## 6. Conclusions

Microalgae represent a sustainable and promising platform for astaxanthin production, yet the industrialization of this high-value ketocarotenoid remains constrained by a series of interconnected bottlenecks, including limited understanding of the regulatory networks governing astaxanthin biosynthesis, suboptimal strain performance, low product titers, high water and energy consumption, and the economic challenges of downstream extraction. This review synthesizes recent advances in strain engineering, metabolic pathway rewiring, cultivation optimization, and green biorefinery technologies, highlighting the critical importance of an integrated, systems-level strategy to overcome these limitations. The development of extremophilic and motile cell-wall-deficient strains, combined with CRISPR-Cas-based genetic editing and multi-omics-guided metabolic reprogramming, has begun to improve astaxanthin biosynthetic efficiency and yield potential in microalgae. Furthermore, the adoption of mixotrophic cultivation, light quality regulation, and Artificial Intelligence (AI)-driven process optimization offers powerful tools to dynamically control carbon flux and enhance astaxanthin productivity. Although microalgal astaxanthin production has advanced considerably, the reported strategies differ greatly in technological maturity. Two-stage *H. lacustris* cultivation, wastewater-coupled production, and supercritical CO_2_ extraction have reached pilot or near-industrial scale, whereas CRISPR-edited strains, extremophilic chassis, and AI-guided metabolic models remain largely at the proof-of-concept or early pilot stage. Therefore, a systems-level framework must not treat these approaches as equivalent. Instead, it should prioritize near-term integration of mature process modules while investing in the scale-up and validation of emerging genetic and computational tools.

In the future, the successful translation of microalgal astaxanthin production into a sustainable bioeconomy will depend on the seamless integration of these approaches into a circular biomanufacturing framework that couples pigment production with wastewater treatment and CO_2_ capture. By advancing both the scientific understanding and technological readiness of these systems, the microalgae-based green astaxanthin intelligent manufacturing paradigm holds great potential to address global challenges in climate change, resource security and sustainable industrial development.

## Data Availability

No new data were created or analyzed in this study. Data sharing is not applicable to this article.
